# Apoptosis regulation by the tyrosine-protein kinase CSK

**DOI:** 10.3389/fcell.2022.1078180

**Published:** 2022-12-12

**Authors:** Andra Fortner, Alexandra Chera, Antoanela Tanca, Octavian Bucur

**Affiliations:** ^1^ Victor Babes National Institute of Pathology, Bucharest, Romania; ^2^ Medical School, Ruprecht-Karls-Universität Heidelberg, Heidelberg, Germany; ^3^ Faculty of Medicine, Carol Davila University of Medicine and Pharmacy, Bucharest, Romania; ^4^ Viron Molecular Medicine Institute, Boston, MA, United States

**Keywords:** apoptosis, Csk, tyrosine-protein kinase CSK, src, Src kinases, SFK, MAPK pathway, PI3K-Akt pathway

## Abstract

C-terminal Src kinase (CSK) is a cytosolic tyrosine-protein kinase with an important role in regulating critical cellular decisions, such as cellular apoptosis, survival, proliferation, cytoskeletal organization and many others. Current knowledge on the CSK mechanisms of action, regulation and functions is still at an early stage, most of CSK’s known actions and functions being mediated by the negative regulation of the SRC family of tyrosine kinases (SFKs) through phosphorylation. As SFKs play a vital role in apoptosis, cell proliferation and survival regulation, SFK inhibition by CSK has a pro-apoptotic effect, which is mediated by the inhibition of cellular signaling cascades controlled by SFKs, such as the MAPK/ERK, STAT3 and PI3K/AKT signaling pathways. Abnormal functioning of CSK and SFK activation can lead to diseases such as cancer, cardiovascular and neurological manifestations. This review describes apoptosis regulation by CSK, CSK inhibition of the SFKs and further explores the clinical relevance of CSK in important pathologies, such as cancer, autoimmune, autoinflammatory, neurologic diseases, hypertension and HIV/AIDS.

## Introduction

Apoptosis defines a programmed form of cell death that leads to caspase activation, controlled specific cellular modifications such as cell shrinkage and formation of apoptotic bodies that are later removed by phagocytes (Li et al., 2021). This serves the homeostasis of multicellular organisms, where old cells are replaced by new ones in order to eliminate degenerated cells, ensure stable tissue size and furthermore plays a critical role during development (Plati et al., 2008; Bucur, 2016). While there are two pathways that initiate apoptosis (Plati et al., 2011), the goal of both is the same: the activation of effector caspases (cysteine-aspartic proteases), enzymes that cleave cellular proteins and activate endonucleases that degrade the DNA in the nucleus (Aitken and Baker, 2013; Bucur, 2016). The extrinsic pathway of apoptosis is initiated by the activation of death receptors such as Fas or death receptors (DR4, DR5), which can polymerize and recruit adaptor proteins when ligands such as FasL and Apo2L/TRAIL are present to trigger the downstream activation of caspase 8 (Kumar et al., 2005; Bucur et al., 2006, 2015; Elmore, 2007). On the other hand, the intrinsic pathway of apoptosis is induced by endogenous DNA damage, which leads to the accumulation of p53, a tumor suppressor protein that acts as a transcription factor for pro-apoptotic genes, e.g., BAX and BAK and many others (Elmore, 2007; Pennarun et al., 2013; Bucur, 2016).

Tyrosine kinases regulate many critical cellular processes and can be divided into two subgroups (Okada, 2012): 1) receptor tyrosine kinases, such as the EGF or VEGF receptors, that typically span the cell membrane and have the ability to autophosphorylate upon ligand binding and dimerization; and 2) cytoplasmic tyrosine kinases, such as CSK and the SRC family tyrosine kinases (SFKs), that can be found in the cytoplasm.

C-terminal Src kinase (CSK) is a cytosolic tyrosine-protein kinase with an important role in regulating critical cellular decisions, such as cellular apoptosis, survival, proliferation, cytoskeletal organization and many others. CSK is an endogenous inhibitor of the SRC family of tyrosine kinases (SFKs) by phosphorylating them on a conserved C-terminal tyrosine residue (Chong et al., 2009). By doing so, CSK regulates important cellular functions, such as cellular apoptosis and survival (Okada, 2012). Since our current knowledge of the CSK mechanisms of action is limited, the known functions that CSK modulates are generally dependent on CSK inhibition of SFKs.

Until now, there are only a few reviews that focused on the tyrosine protein kinase CSK. For instance, Okada gives a great overview over the structure, function and discovery of CSK (Okada, 2012). Also, the review by Kim et al. (Ia et al., 2010) and a different review by Chong et al. (Chong et al., 2009) focused on the interaction of SFKs with CSK-family kinases, describing the structural inactivating mechanism CSK exerts on SFKs.

In this Review, we are focusing on revealing the apoptosis regulation mechanisms by CSK, CSK inhibition of the SFKs, while further exploring the clinical relevance of CSK in important pathologies, such as cancer, autoimmune, autoinflammatory, neurologic diseases, hypertension and HIV/AIDS.

## SRC family of tyrosine kinases

SFKs are a family of cytoplasmic tyrosine kinases that can be found in mammalian cells (Dodd et al., 2014).

They can be activated by different transmembrane receptors, such as the EGF receptor, T cell receptor or integrins and constitute an integral part of signaling cascades regulating cell proliferation, differentiation and cell motion (Thomas and Brugge, 1997).

There are currently ten known types of SFKs (c-Src, c-Yes, Fyn, c-Fgr, Lyn, Hck, Lck, Blk, Frk, Hck), c-Src, c-Yes and c-Fgr being the human analogues of the historically earlier discovered viral SFKs (Okada, 2012; Amata et al., 2014). Some SFK types, such as c-Src, Yes and Fyn, can be ubiquitously found, while other SFK types are only expressed in specific cell types. For instance, Lck is particularly located in T-cells and plays a critical role in lymphocyte T-cell activation (Engen et al., 2008; Manz et al., 2015).

**FIGURE 1:**
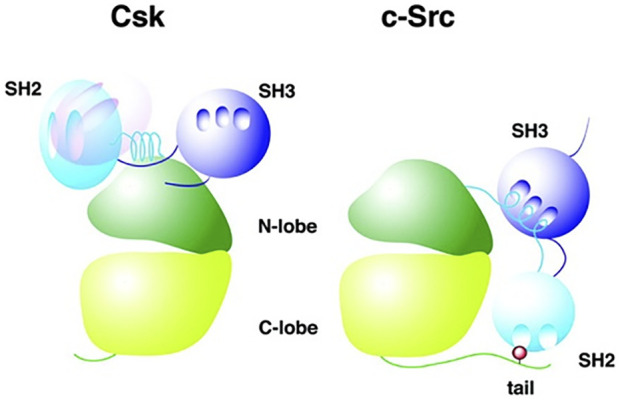
Comparison of CSK and SFK structure. CSK (left) and SFK (right) structures are illustrated, showing the configuration of the respective domains of the two molecules. Here, c-Src is used as a representative for SFKs. The hollows indicate binding sites in the SH2 and SH3 domain. The light blue SH2 domain of CSK (left) represents its location in an active state of the CSK molecule, whereas the pink SH2 domain shows SH2 domain position in an inactive molecule. The red dot in the C-terminal domain of c-Src (right) shows the C-terminal regulatory tyrosine (Tyr-527) where CSK phosphorylates c-Src in order to inhibit c-Src. Adapted from Reference (Ogawa et al., 2002), with permission, under the Creative Commons Attribution (CC BY 4.0) License.

SFKs exist in an active and inactive state, depending on the three-dimensional phenotype of the protein. In order to understand SFK function and regulation, it is helpful to visualize the general structure of SFKs (Figure 1 uses c-Src as a representative SFK).

c-Src (as an example for SFK) can be divided into the following functional domains (Okada, 2012):

• N-terminal domain (also called SH4): it contains an acylation site (myristoylation or palmitoylation) responsible for plasma membrane adherence of SFKs.
• unique domain: this domain differs among the different SFK types and determines functional specificity (Sirvent et al., 2012; Amata et al., 2014; Ortiz et al., 2021).
• Src homology domain 3 (SH3)
• Src homology domain 2 (SH2)
• tyrosine kinase domain (also called SH1): it contains an ATP-binding site, a binding site for the target protein and two loops in the cleft between both. The activation loop includes an autophosphorylation-loop with regulatory function and a catalytic loop (Lin et al., 2003).
• C-terminal domain:it contains a tyrosine residue that carries out a regulatory function upon phosphorylation.

In order to inactivate a SFK, a tyrosine residue at the C-terminus of the SFK can be phosphorylated by kinases, causing an intramolecular interaction of the tail with the SH2 domain (Engen et al., 2008). In addition, an interaction between the SH3 domain and an amino acid sequence connecting the SH2 with the tyrosine-kinase domain is formed (Engen et al., 2008). These changes in protein conformation downregulate SFK activity.

In contrast, SFK activation can be achieved by autophosphorylation of one or more conserved tyrosine residues in the autophosphorylation-loop, in the kinase domain (Lin et al., 2003; Chong et al., 2005). Simultaneously, phosphatases dephosphorylating the C-terminal tyrosine residue are needed as well, to undo the conformation change that occurs when inactivating the SFK.

**FIGURE 2:**
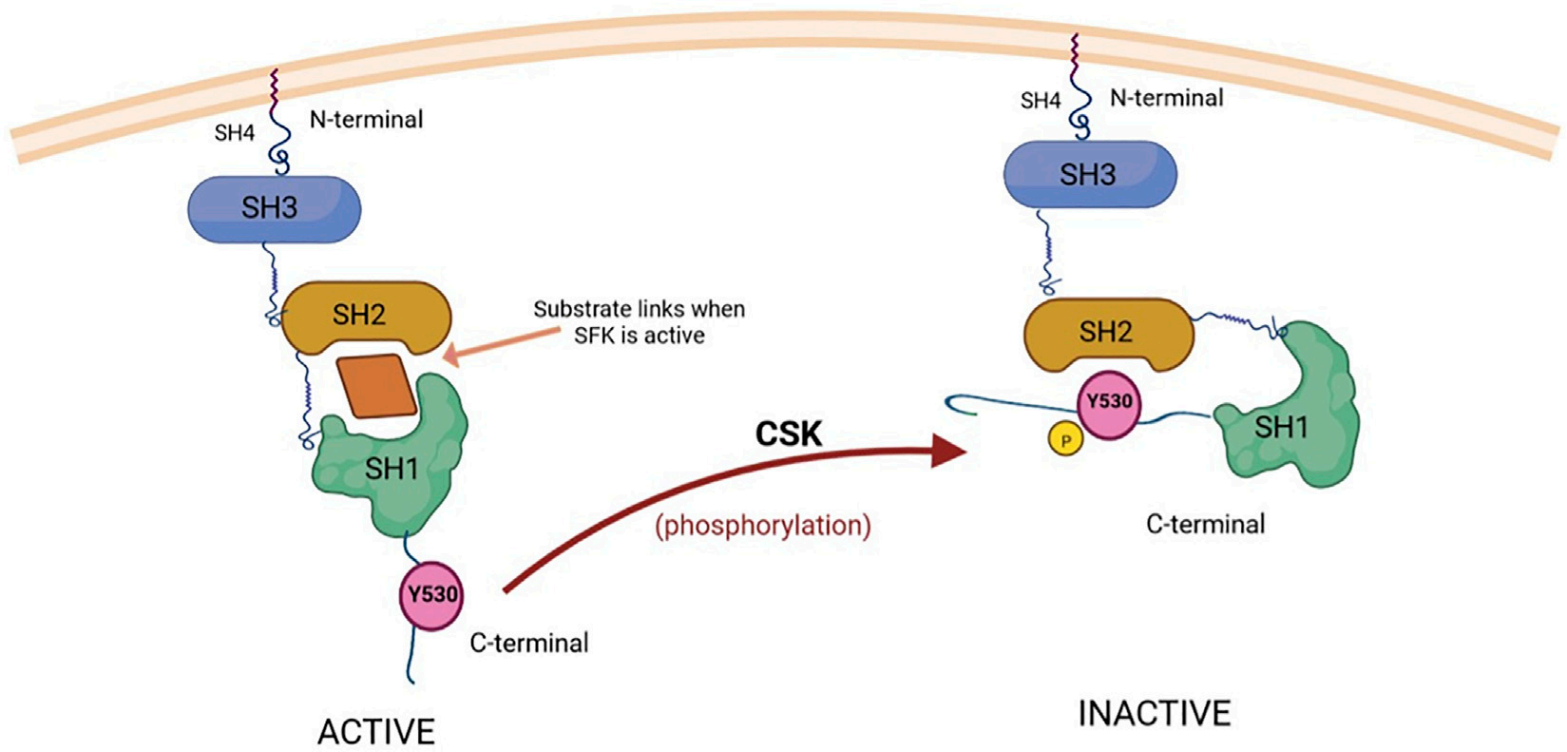
CSK-mediated inactivation of substrate kinases (SFKs) through phosphorylation. The substrates of CSK, Src proteins (SFKs), are composed of three domains (SH1, SH2 and SH3), with an additional SH4 domain situated on the N-terminal region, which has an important role in attaching Src to the cell membrane (Kim et al., 2009). SH1 is the catalytic domain responsible for the tyrosine kinase activity of Src, while SH2 and SH3 can interact with other signaling proteins. The regulation of Src activity is ensured through phosphorylation and dephosphorylation of a C-terminal tyrosine residue (Y530) (Chen et al., 2014). The active form (on the left) is able to bind substrates while Y530 is not phosphorylated. When the C-terminal Src kinase (CSK) phosphorylates Y530, the conformation of Src changes and it becomes unable to bind substrates and exert its tyrosine kinase activity (the inactive form, shown on the right) (Reinehr et al., 2013; Ortiz et al., 2021). Created with BioRender.com.

Regarding SFK regulation at the C-terminal tyrosine, phosphorylation can be carried out by C-terminal Src kinase (CSK) (Figure 2) or by CSK homologous kinase (CHK), whereas phosphatases identified to remove the phosphate group from the tyrosine can be found in the cytoplasm (PTP1B, Shp1, Shp2) or in the plasma membrane, where they reside as transmembrane enzymes (CD45, PTPɑ, PTPγ, PTPε) (Roskoski, 2005).

In addition to this catalytic SFK regulation mechanism, SFKs can also be regulated by non-catalytic binding of an inhibitor to the SFK protein. Examples for such non-catalytic inhibitors are CHK, WASP, caveolin and RACK1 (Chong et al., 2005).

Meticulous control of SFK activity is very important in order to ensure proper receptor signaling. Moreover, increased activation of SFK has an oncogenic effect, and thus it is associated with cancer development and progression (Guarino, 2010). T cells from mice with a mutated CSK, displayed longer and elevated T-cell receptor signaling and stronger T-cell proliferation (Manz et al., 2015). Similarly, IgE receptor-mediated mast cell signaling and degranulation increased when knocking down CSK, while simultaneously lessening production of proinflammatory cytokines (Potuckova et al., 2018).

## Tyrosine protein kinase CSK

C-terminal Src kinase (CSK) is an endogenous inhibitor of SFKs by phosphorylating the C-terminal tyrosine residue of the SFK, as described above (Sondhi et al., 1998).

A CSK molecule weighs 50 kDa and is built of three functional domains: SH3, SH2 and kinase domain (Okada, 2012). In comparison to SFK structure, CSK does not contain an N-terminal acylation group and the regulatory tyrosine residues in the kinase and C-terminal domain. Furthermore, SFKs and CSK differ in the position of the SH2 domain (Figure 1) (Roskoski, 2004).

As CSK, unlike SFKs, does not contain an acylation site, it is a cytoplasmic protein and hence needs to be recruited to the plasma membrane in order to exert its inhibitory function (Manz et al., 2015; Potuckova et al., 2018). Therefore, adaptor proteins are needed. Csk-binding protein (Cbp), also called phosphoprotein associated with glycosphingolipid microdomains (PAG), is such a transmembrane protein that can be found in lipid rafts (Davidson et al., 2003). In T cells, Cbp binds the SH2 domain of CSK, which inhibits SFKs (Potuckova et al., 2018). When Cbp gets dephosphorylated, e.g. upon activation of a T-cell receptor, it dissociates from CSK, thus enabling SFK activity, since the SFKs are no longer inhibited by CSK (Torgersen et al., 2001). Other adaptor proteins include Lck-interacting molecule (LIME) (Brdičková et al., 2003), paxillin (Schaller and Parsons, 1995), Dok-1 and Dok-2 (Yasuda et al., 2007), VE-cadherin (Baumeister et al., 2005), caveolin-1 (Lee et al., 2000) and ZO-1 (Saito et al., 2008).

Similarly, CSK has been found to bind to the protein-tyrosine phosphatases (PTP) PEP, PTP-PEST and PTP-HSCF in hematopoietic cells, whereby association of CSK with PTP-PEST can also be observed in non-hematopoietic cells (Davidson et al., 1997; Wang et al., 2001). Whereas PEP and PTP-PEST use a proline-rich region outside the catalytic region to attach to the SH3 domain of CSK, PTP-HSCF binds to the SH2 domain of CSK (Cloutier and Veillette, 1996; Davidson et al., 1997; Wang et al., 2001). The complex of CSK with a PTP serves a more powerful inhibition of SFKs. On the one hand, PTPs exert their dephosphorylation of the tyrosine in the activation loop to inactivate the SFK (Cloutier and Veillette, 1999), and on the other hand, binding of a PTP to a CSK improves CSK function (Wang et al., 2001). Thus, PTPs do not only serve as recruiters of CSK to activated SFKs, but simultaneously support the inhibitory function of CSK.

Recently, Cui et al. found that CSK expression can be altered by SUMOylation, which is a post-translational modification of proteins that occurs in cells, where a small ubiquitin-like modifier (SUMO) is attached to a lysine of the target protein by the SUMO E1, E2 and E3 enzymes (Cui et al., 2019). CSK is SUMOylated on lysine 53, which negatively affects its function (Cui et al., 2019). Thus, less CSK is recruited to the lipid rafts after SUMOylation, which comes along with an increase in SFK activity (Cui et al., 2019).

Regarding the regulation of the CSK-SFK interaction, a negative feedback exists, activating CSK during SFK hyperactivity, although further investigation is required to elucidate the mechanism in detail (Jiang et al., 2006; Chen et al., 2014).

Furthermore, apart from its inhibitory function of SFKs, CSK has been observed to play an important role in gliotactin protein regulation, by promoting its endocytosis independently from its effect on SFKs (Samarasekera and Auld, 2018). Gliotactin is a protein that can be found at tricellular junctions, i.e. the location where tight junctions from three epithelial cells meet. There, it is responsible for orderly cell adhesion. Hence, CSK participates in maintaining cellular structure in a pathway that does not require SFKs, but of course, signaling cascades involving SFKs are critical for proper cell morphology. Inactivation of CSK in squamous epithelia in mice led to a compromise in cell-cell attachment, because of a change in cytoskeletal organization, which was mediated through CSK’s effect on SFKs (Yagi et al., 2007).

## Tyrosine-protein kinase CSK regulation of apoptosis and other cellular functions

**FIGURE 3:**
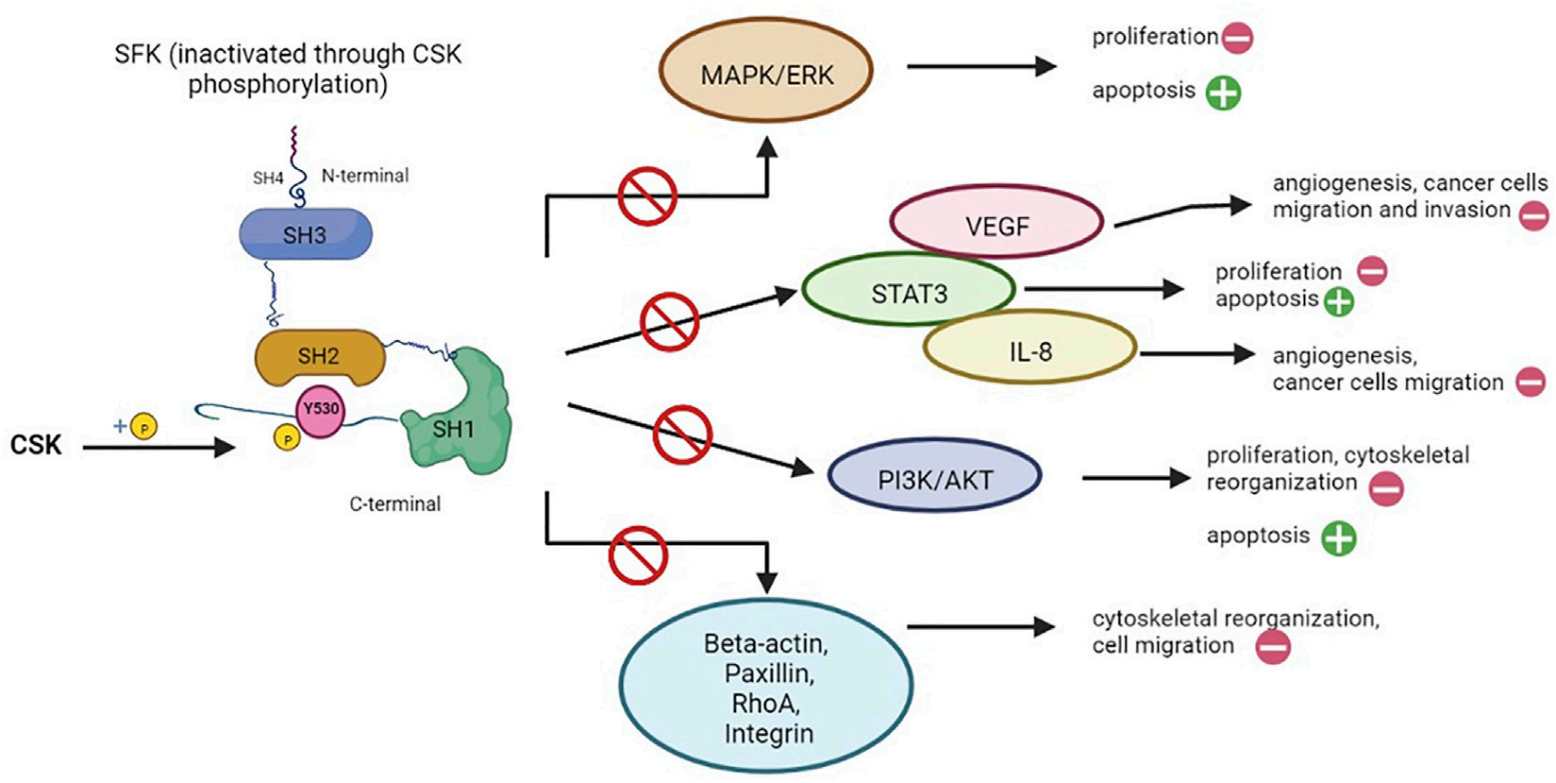
Cellular functions regulated by CSK through inhibition of SFK signaling pathways. Src family proteins (SFKs) are involved in a multitude of signaling pathways, mostly responsible for cell proliferation (e.g. MAPK/ERK, STAT3, PI3K/AKT pathways), angiogenesis (e.g., VEGF, IL-8) and cytoskeletal reorganization. Through phosphorylation (as seen on the left), CSK is able to inactivate SFKs, thus exerting an inhibitory effect on all the pathways in which the latter is involved (represented on the right). The main results are: promoting apoptosis (as opposed to cell proliferation), inhibition of angiogenesis, interrupting the pro-oncogenic effects of SFK such as cancer cell migration and invasion, while also interfering with cytoskeletal reorganization (Kim et al., 2009; Chen et al., 2014). Created with BioRender.com.

**FIGURE 4:**
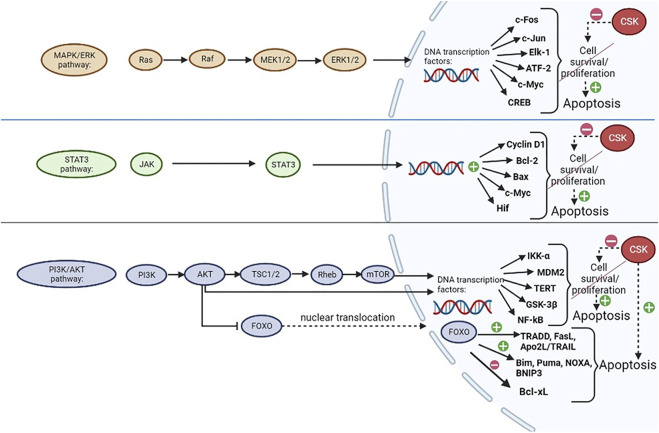
CSK regulation of apoptosis. CSK inhibits MAPK/ERK, STAT3 and PI3K/AKT signaling pathways, which are normally involved in promoting cell proliferation and/or survival, through regulation of transcription factors in the nucleus, thus preventing their augmentative effects on cell growth and promoting apoptosis. CSK could potentially promote apoptosis through activation of Forkhead box, subclass O (FOXO) proteins, which are usually inactivated by AKT when the PI3K/AKT pathway is active. FOXO has upregulating and downregulating effects on many transcription factors, ultimately promoting apoptosis (Dumitrascu and Bucur, 2013; Fang et al., 2013; Bucur et al., 2014; Chen et al., 2014; Guo et al., 2020; Ma et al., 2020; Vidal et al., 2021). Created with BioRender.com.

CSK regulates a wide variety of cellular functions, such as apoptosis, proliferation, cytoskeletal reorganization, cell migration, invasion, angiogenesis, mainly through the modulation of SFKs, while also being involved in preventing cancer progression, since SFKs have been associated with cell differentiation, cancer stage and development of metastases (Kim et al., 2009; Chen et al., 2014). As illustrated in Figures 3, 4, these functions are modulated and controlled by the influence exerted on specific pathways, such as mitogen-activated protein kinase (MAPK)/extracellular signal-regulated kinases (ERK) pathway, signal transducer and activator of transcription 3 (STAT3) pathway and phosphatidylinositol-3-kinase (PI3K)/AKT signaling pathway, alongside the direct influence over other specific substrates and/or pathways, such as beta-actin, paxillin, rhoA or integrins, mostly involved in cytoskeletal organization and other functions (Chen et al., 2014).

The main results of these interactions are: stimulating apoptosis (as opposed to cell survival), inhibition of angiogenesis, interrupting the pro-oncogenic effects of SFKs, such as cancer cell migration and invasion, while also interfering with cytoskeletal reorganization (Figure 3) (Kim et al., 2009; Chen et al., 2014).

As shown in Figure 4, the three main signaling pathways that are heavily influenced by SFKs in a positive manner (promoting cell survival/proliferation) as opposed to the negative influence of CSK which could promote apoptosis (by preventing the effects of SFKs) are MAPK/ERK, STAT3 and PI3K/AKT pathways (Chen et al., 2014).

The MAPK/ERK enzymatic cascade is crucial for cellular division. ERK itself is a member of the MAPK family with mitogenic abilities provided by regulating transcription factor activity and gene expression in the cell nucleus. The effects of this pathway are ensured by the activation of ERK, which is achieved through an activating sequence starting from Ras and continuing with the activation of Raf, MEK1/MEK2 and subsequently, ERK. When the latter is activated, it migrates from the cytoplasm to the nucleus, phosphorylating various transcription factors, such as c-Fos, c-Jun, Elk-1, ATF-2 and gene expression regulators such as CREB and c-Myc, in order to achieve its functions (Chen et al., 2014; Guo et al., 2020).

The STAT3 signaling pathway, which is a JAK-STAT pathway, is initiated when ligands, such as cytokines (interferons and interleukins; e.g. interleukin-6, involved in breast cancer progression) bind to its receptor which is coupled with a Janus kinase (JAK) that further activates STAT transcription factors, such as STAT3, through phosphorylation. This will further activate the expression of cyclin D1, Bcl-2, Bax, c-Myc, hypoxia-inducible factor (Hif) and other genes, therefore stimulating cell growth and ensuring increased cell survival (Chen et al., 2014; Ma et al., 2020).

The PI3K/AKT pathway is initiated when a growth factor binds to a tyrosine kinase receptor, thus activating PI3K which is able to phosphorylate AKT. AKT further phosphorylates TSC2 (within a TSC1/TSC2 dimer), stimulating Rheb and ultimately activating mTOR (Fang et al., 2013). Both mTOR and AKT have the ability to regulate protein synthesis in the nucleus in order to promote cell division, by modulating the activity of IκB kinase alpha (IKK-α), MDM2, telomerase reverse transcriptase (TERT), glycogen synthase kinase-3β (GSK-3β) and nuclear factor κB (NF-κB) (Chen et al., 2014; Vidal et al., 2021). AKT also phosphorylates and inactivates Forkhead Box O (FOXO) transcription factors proteins, which will remain sequestrated in the cytoplasm. When the PI3K/AKT pathway is inhibited (by CSK, for example), FOXO family of transcription factors could potentially be activated through dephosphorylation, becoming able to translocate to the nucleus and regulate the expression of various genes involved in the control of apoptosis, proliferation, cell differentiation and many other critical cellular processes (Singh et al., 2010; Bucur et al., 2014). Through this mechanism, FOXOs can promote apoptosis, which can be initiated either by the intrinsic pathway, if FOXO proteins upregulate pro-apoptotic members of the Bcl-2 family (such as Bim, Puma, NOXA, BNIP3) and downregulate the expression of the anti-apoptotic members of the Bcl-2 family (such as Bcl-xL), or by the extrinsic pathway through upregulation of TRADD, FasL, Apo2L/TRAIL, cFLIP and other proteins (Dumitrascu and Bucur, 2013). FOXO proteins are also involved in cell cycle arrest by upregulating p21 and p27 expression, while also downregulating cyclin D1 and cyclin D2 expression (Bucur et al., 2014).

Furthermore, inhibition of mTOR by modulation of SFK activity is not only a stimulator for apoptosis, but also for autophagy. For example, tumor-suppressing STF cDNA 3 (TSSC3) can interact with an SFK-bound RanBP9 protein, thereby inhibiting SFK function and the PI3K/AKT pathway, and thus promoting apoptosis and autophagy (Dai et al., 2016; Zhao et al., 2018). Loss of TSSC3 expression has been found to be involved in the pathogenesis of different cancers, e.g. osteosarcoma (Dai et al., 2012). However, increased SFK activity can also lead to autophagy. That is the case with interleukin IL-17A expression, that arises in neural tissue under ischemic conditions (Liu et al., 2019). SFKs get activated by IL-17 receptors, negatively regulating mTOR *via* PP2B activation to induce autophagy (Liu et al., 2019). Interestingly, SFKs can be targeted to influence deregulated autophagy in diseases. For instance, Kim et al. described 2-cyano-3,12-dioxo-oleana-1,9(11)-dien-28-oic acid methyl ester (CDDO-Me), a substance that activates nuclear factor-erythroid 2-related factor 2 (Nrf2), to also be able to inhibit SFKs and the PI3K/AKT pathway in order to regulate autophagy and clasmatodendrosis in astrocytes in the epileptic hippocampus (Kim and Kang, 2021).

Another interesting aspect of apoptosis regulation by CSK is a change in actin dynamics in order to enable morphologic changes of cells during apoptosis, leading to the formation of apoptotic bodies which can be removed by phagocytes (Bucur et al., 2001). Notably, apoptosis comes with dramatic changes in the organization of the cytoskeleton (Franklin-Tong and Gourlay, 2008; Lavoie et al., 2010): whereas microtubules and intermediate filaments dissemble, actin dynamics undergo huge changes that include actin formation, as well as actin destruction in the course of apoptosis.

Interestingly, in a study using transforming growth factor (TGF)-beta1 to stimulate the extrinsic apoptosis pathway, SFK activity was observed to shortly increase before dramatically declining (Park et al., 2004). Although not investigated in the study, this might suggest a delayed inhibition of SFKs by CSK, which eventually could lead to SFK cleavage into fragments that are translocated to the nucleus, as described by Park et al. (Park et al., 2004).

Since SFK activation plays an important role in regulating actin polymerization and thus, intracellular transport of proteins, initially increasing SFK activity might trigger a sudden change in actin organization, resulting in deranged traffic in the cell (Peterson and Chernoff, 2006; Bharti et al., 2007; Lavoie et al., 2010). These effects are obtained through phosphorylation of small GTP-binding proteins from the Rho family (e.g. RhoA, Rac1 and Cdc42) by SFKs, that in turn initiate other signaling cascades that regulate the disruption of cytoskeletal dynamics (Robert et al., 2006):

• RhoA activates Rho-associated protein kinase (ROCK), that among others is able to phosphorylate myosin light chain (MLC) contributing to contraction of actin-myosin and the formation of stress fibers, or it can for instance phosphorylate LIM kinase, which leads to an inhibition of actin depolymerization because of inhibited cofilin activity (Hartmann et al., 2015).
• Upon phosphorylation by SFKs, Rac1 is increasingly recruited to focal adhesions and Rac-guanine nucleotide exchange factors (Rac-GEFs) (Chang et al., 2011; Cooke et al., 2020). Rac-GEFs promote the nucleotide exchange of GDP to GTP in order to activate Rac. Stimulation of Rac-GEFs can be obtained by interaction of the Rac-GEF with receptor tyrosine kinases through Gab1-Grb2 adaptors, a mechanism that plays an important role in developing cell motility, especially in lung cancers (Kazanietz et al., 2022). Activated Rac1 can then stimulate the diaphanous-related formin (DRF) FHOD1 which can regulate actin stress fiber and microtubule arrangement, lamellipodia formation while also exerting its function on the transcription of specific genes (Gasteier et al., 2003, 2005). Cleavage of ROCK1 by caspases results in a constitutive active form of the kinase, which contributes to the typical alterations in cell structure for apoptosis (Coleman et al., 2001). Both, FHOD1 and ROCK1 cooperate to induce the formation of plasma membrane blebs, which especially arise when a cell is undergoing apoptosis (Hannemann et al., 2008).
• Cell division control protein 42 homolog (Cdc42) plays a role in many cellular processes, one of these being actin formation (Cao et al., 2022). When activated, for example by SFKs, Cdc42 binds to a protein of the Transducer of Cdc42 dependent actin assembly (TOCA) family, which links Cdc42 to Wiskott-Aldrich syndrome protein (WASP) (Ho et al., 2004; Watson et al., 2017). This complex is able to activate Arp2/3, which triggers the polymerization of new actin fibers (F-actin) (Cao et al., 2022).

The subsequent decrease in SFK activity mediated by CSK is necessary to suppress survival signaling of the apoptotic cells as described above. Hence, CSK cleverly influences SFK activity to ensure the proper sequence of cellular events needed for apoptosis.

## Clinical importance of tyrosine-protein kinase CSK

Aberrant CSK and/or SFK activity can be associated with a large number of diseases, such as cancer, autoimmune, autoinflammatory and neurologic diseases, hypertension and HIV/AIDS (Engen et al., 2008; Szilveszter et al., 2019; Xu et al., 2021). This highlights the importance of CSK as a key regulator of SFKs and its potential as a therapeutic target in the treatment of these diseases.

### Cancer

As SFKs are involved in signaling cascades for cell survival, proliferation and migration, it is no surprise that anomalous SFK activity promotes tumorigenesis. Indeed, high levels and activity of SFKs have been reported in different types of tumors, amongst others lung, skin, colon and breast cancer (Ishizawar and Parsons, 2004). For example, Redin et al. have demonstrated that Yes1, a member of the SFKs, is involved in increasing the number of regulatory T-cells (Treg) which are able to infiltrate tumors in cases of non-small cell lung cancer (NSCLC), thus Yes1 being one of the most important predictors of a poor prognosis in this type of cancer. Subsequently, they have tried using SFK inhibitors in this particular type of tumor alongside immune checkpoint inhibitors (ICIs) which were already known to have a beneficial effect, resulting in accelerated tumor regression compared to monotherapy, as the SFK inhibitor dasatinib has acted synergically with the ICIs while also decreasing the number of Tregs (Redin et al., 2021). This points out a possible function of CSK as a tumor suppressor as well. Masaki et al. observed reduced CSK levels in hepatocellular carcinoma of humans and rats, indicating such an anti-oncogenic effect of CSK (Masaki et al., 1999). However, the extent and exact mechanism of how CSK contributes to tumor suppression is being disputed, because of the following reasons (Sirvent et al., 2012): 1) CSK extracted from colorectal cancer cells remains functional in terms to its SFK inhibition when tested *in vitro* (Sirvent et al., 2012). 2) CSK is a tumor antigen, as it can generate autoantibodies in patients with carcinoma (Sirvent et al., 2012). 3) no mutations in the CSK gene modifying CSK function are known so far (Okada, 2012; Sirvent et al., 2012). 4) most interestingly, CSK is not always downregulated in malignant cells that exhibit increased SFK activity. In other words, CSK levels have sometimes been observed to increase along with SFK activity, but CSK in these cases has non-etheless been unable to inhibit SFK function and thus tumorigenesis (Watanabe et al., 1995). Instead, Cbp has been identified as the key regulator of whether CSK can actually exert its inhibitory function on SFKs or not, because it is essential for recruitment of CSK to the plasma membrane as described above (Sirvent et al., 2009). Accordingly, Cbp levels are usually downregulated in tumors with elevated SFK function. Re-expressing Cbp in these tumors leads to a halt in tumor development (Oneyama et al., 2008). SFKs have been shown to repress Cbp expression on the transcriptional level, resulting in even greater SFK function, because of the impairment of the inhibitory mechanism using CSK (Oneyama et al., 2008). Moreover, Cbp can also be downregulated by the oncogenic small G-protein Ras (Oneyama et al., 2008). Nevertheless, elevated CSK levels might have a tumorigenesis-promoting effect *via* another signaling pathway: CSK has been shown to phosphorylate eukaryotic elongation factor 2 (eEF2), a protein that is usually important for the translation during protein expression in the cytoplasm (Yao et al., 2014). Phosphorylation by CSK does not have any effects on eEF2 function. However, it encourages decomposition of eEF2 into small fragments, enabling these fragments to translocate into the nucleus. SUMOylation is also associated with this phenomenon. eEF2 fragments in the nucleus alter nuclear structure and trigger aneuploidy. The suggestion that this mechanism contributes to tumorigenesis is in line with the finding that many tumors display elevated levels of eEF2 (Chen et al., 2011).

Interestingly, it has also been shown that CSK may be directly inhibited in some tumors. In this regard, Sun et al. found epidermal growth factor, latrophilin and seven transmembrane domain-containing protein 1’s (ELTD1) expression is heightened in gastric cancer. ELTD1 is a G-protein-coupled receptor usually playing a role in angiogenesis, cellular metabolism and cardiac hypertrophy (Sun and Zhong, 2021). In tumors, interaction of ELTD1 with CSK inhibits CSK function, thus promoting MAPK/ERK signaling which leads to increased cell proliferation, epithelial to mesenchymal transition and metastasis (Sun and Zhong, 2021).

To sum up, CSK is effective in suppressing cancer development, whereas its downregulation supports tumorigenesis. Thus, dysfunction of the mechanisms that ensure proper CSK function in a normal cell, promotes cancer.

### Autoimmune disease

Apoptosis is an essential process in many aspects of immune functions, and it is known that defects in apoptosis can lead to immune system impairment by developing autoimmunity (Feig and Peter, 2007). Seeing that SFKs and CSK are deeply involved in the regulation of apoptosis, it is implied that they can also influence immune processes. For example, the deletion of Lyn (a member of the SFKs family) in mature follicular B cells has been proven to induce resistance to apoptosis, therefore promoting proliferation and differentiation of antibody-producing B-cells and inducing a positive effect on the immune response, while it is also known that the same protein is involved in both positive and negative regulatory pathways (Brian and Freedman, 2021). The tyrosine-protein kinase CSK has been associated with systemic lupus erythematosus (SLE). When an antigen stimulates an immune cell by binding to the B-cell receptor (BCR) or the T-cell receptor (TCR), SFK activity is regulated by the kinase CSK and by phosphatases in opposite directions (Manjarrez-Orduño et al., 2012; Hui and Vale, 2014). Lymphoid tyrosine phosphatase (Lyp) is such a phosphatase that is particularly found in hematopoietic cells. It is encoded by the protein tyrosine phosphatase non-receptor 22 (PTPN22) gene, where single nucleotide polymorphisms (SNP) (C1858T, rs2476601) have been reported as a risk factor for autoimmunity (Tizaoui et al., 2021). Since CSK and Lyp form a complex in developing immune cells and because Lyp variants have been shown to escape interaction and association with CSK, it has been postulated that not only SNPs in Lyp but also in CSK might be responsible for the development of autoimmune disease (Manjarrez-Orduño et al., 2012). Manjarrez-Orduño et al. found the variant rs34933034 associated with SLE and situated in an intronic regulatory region of CSK to increase CSK expression in B-cells. The elevated CSK levels lead to a stronger inhibition of Lyn, a SFK that - unlike other SFK members - inhibits immune signaling pathways (Manjarrez-Orduño et al., 2012; Brian and Freedman, 2021). Thus, the activation of immune cells carrying the rs34933034 variant is greater than in cells without it (Manjarrez-Orduño et al., 2012). Furthermore, B cell precursors exhibit higher levels of CSK than in a more adult stage (Manjarrez-Orduño et al., 2012). The probability of an increased number of autoreactive B-cells that might emerge due to a dysfunction of a tolerance checkpoint is higher in carriers of the risk allele, resulting in production of autoantibodies (Manjarrez-Orduño et al., 2012). This is observed in autoimmune diseases, such as SLE. Overall, CSK has been shown to attenuate activation of immune cells, thus proposing CSK as potential therapeutic target (Manz et al., 2015).

### Autoinflammatory disease

In contrast to autoimmune diseases where the specific/acquired immune system is impaired, the innate immune system is affected in autoinflammatory diseases. Autoinflammatory disease is characterized by systemic inflammation in the body, resulting from excessive cytokine production and apoptosis (Xu et al., 2021). CSK has been reported to play an important role in the pathophysiology of these illnesses.

Proline-serine-threonine-phosphatase-interacting protein 2 (PSTPIP2) which can especially be found in macrophages, is a protein of the Fes/CIP4 homology-Bin/Amphiphysin/Rvs (F-BAR) family that regulates membrane structure, by forming indentations in the membrane (Liu et al., 2015). While binding to the membrane with its N-terminal domain, PSTPIP2 is able to attract PTP-PEST to its C-terminal domain, thereby recruiting CSK, which then interacts with PSTPIP2 (Xu et al., 2021). Apart from CSK, Src homology domain-containing inositol 5’-phosphatase 1 (SHIP1) is another negative regulator of PSTPIP2 (Drobek et al., 2015). Inhibition of PSTPIP2 causes a stronger response to colony-stimulating factor 1 (CSF-1) than usual, also called macrophage colony-stimulating factor (M-CSF), by stimulating the serine/threonine kinases ERK1/2 (extracellular-signal regulated kinases) and STAT1 (Xu et al., 2021). ERK-signaling is involved in macrophage proliferation and differentiation (Richardson et al., 2015). This leads to inflammation, promoting autoinflammatory diseases (Xu et al., 2021). Hence, although PSTPIP2 is being discussed as the key protein in the development of autoinflammatory diseases, CSK nevertheless plays an important role in regulating the extent of an inflammation.

There are also members of SFKs that are involved in cytotoxicity mediated by T-cells and natural killer cells. One such protein is Fyn, which is an important regulator of cytokine production and cytotoxicity through the Fyn-phosphoinositide 3-kinase (PI3K) pathway and the Fyn-adhesion and degranulation-promoting adaptor protein (ADAP) axis (Gerbec et al., 2015).

### Neurologic disease and brain development

Elevated protein levels of Fyn, an SFK, have been observed in several neurologic diseases, such as Alzheimer’s disease and epilepsy (Kojima et al., 1998; Chin et al., 2005). Normally, Fyn activity is essential for various processes in the CNS, such as synaptic plasticity, myelin production, synaptic signaling and neuronal migration during development (Knox and Jiang, 2015). In neurons, Fyn is located at the postsynaptic membrane of synapses, where it interacts with N-methyl-D-aspartate (NMDA) receptors, in order to promote their open state and enhance the Ca2+ influx (Salter and Kalia, 2004; Trepanier et al., 2012). Fyn is involved in the development of Alzheimer’s triggering synaptotoxicity when expressed with amyloid-β (Aβ) peptides in mice (Chin et al., 2005). It also contributes to neuronal hyperexcitation, causing epileptic seizures (Kojima et al., 1998; Anwar et al., 2020; Putra et al., 2020). Aβ gets deposited as amyloid plaque in the brain as an early sign of Alzheimer’s, whereas neurofibrillary tangles containing the protein tau form as the disease progresses (Williamson et al., 2002; Briner et al., 2020). Notably, Fyn overexpression accelerates neural mortality and memory deficits in mice expressing moderate Aβ levels, in a way that is usually seen in mice with significantly higher Aβ levels (Chin et al., 2005). By hyperphosphorylating tau protein at Tyr-18, Fyn contributes to formation of neurofibrillary tangles and thus exacerbates clinical symptoms (Briner et al., 2020; Liu et al., 2020). Being an inhibitor of Fyn, CSK might be important in the development of these neurologic diseases. However, no studies regarding CSK’s role in these diseases have been published so far and further research is needed to determine CSK’s contribution to neurodegeneration and epilepsy.

Furthermore, CSK has also been shown to play a critical role during development. On the one hand, deletion of the CSK gene in mice caused lethal dysfunction of the neural tube and necrosis (Imamoto and Soriano, 1993; Nada et al., 1993). Another group studying the effect of constitutively active SFKs in the developing eyes of drosophila found that proliferation and apoptosis increased compared to their control group (Pedraza et al., 2004). On the other hand, CSK overexpression leads to abnormal and reduced growth of neurites (Dey et al., 2005, 2007). Also, mice mutant for Fyn showed abnormal hippocampal formation with functional impairments of long-term potentiation and learning (Grant et al., 1992). These findings indicate that neither too much nor too less SFK activity is bearable during neurologic development. Interestingly, the amount of CSK in cells differs in the developing and in the adult brain: CSK levels in the brain decreases during development (Lindquist et al., 2011). This suggests an increase in SFK activity at the same time, which might be necessary to coordinate proper cell growth and differentiation. However, it has been suggested that there might also exist an additional regulatory mechanism for SFKs that keeps their activity in check as development progresses (Lindquist et al., 2011). Still, CSK is absolutely critical to ensure proper development of the nervous system.

### Hypertension

Blood pressure is controlled by various mechanisms, including short- medium- and long-term mechanisms (Ahmed et al., 2020; 2021; de Bhailis and Kalra, 2022; Tomaszewski et al., 2022). CSK has been shown to engage in blood pressure regulation and CSK depletion has been identified as a possible trigger for hypertension (Lee et al., 2016; Oh et al., 2018). Low CSK levels result in diminished inhibition of SFKs which leads to enhanced transcription of the cytochrome P450 family 11 subfamily B member 2 (CYP11B2) gene. CYP11B2 codes for a 18-hydroxylase that is needed for aldosterone synthesis in the zona glomerulosa of the adrenal gland (Gomez-Sanchez et al., 2022). Thus, CSK deficiency triggers augmented aldosterone production, which in turn increases sodium reabsorption in the kidney, *via* serum and glucocorticoid-inducible kinase-1 (SGK1) (Oh et al., 2018; Valinsky et al., 2019). Interestingly, simultaneous exposure of vascular smooth muscle cells (VSMCs) to aldosterone and angiotensin II (Ang II) has a potentiating effect in triggering vasoconstriction (Rautureau et al., 2011). In the kidney, SGK1 mediates a rise in sodium reabsorption, by activating the epithelial sodium channel (ENaC), the NaCl cotransporter (NCC) and Na+/H+ exchanger 3 (NHE3) (Satoh et al., 2015). However, it also takes part in potassium homeostasis by inhibiting the renal outer medullary potassium channel (ROMK), through a pathway that involves WNK1 (With No Lysine 1; lysine = K). This leads to water retention, promoting higher blood pressure. The reason for reduced CSK activity in these cells might be Ang II signaling, since it has been shown in VSMCs of Spontaneously Hypertensive Rats (Touyz et al., 2002).

All in all, the increase in sodium levels and thus plasma volume on the one hand, and the disturbing influence of Ang II and aldosterone on VSMCs on the other hand, that are both mediated through a decrease in CSK activity, promote hypertension.

### HIV/AIDS

Over 650.000 people died of AIDS and ∼1,5 million people were infected with HIV in the year 2020 alone (Fortner and Bucur, 2022). Nef is a protein produced by the human immunodeficiency virus 1 (HIV-1) and it plays an important role in the virulence and development of the disease (Harris, 1999; Alvarado et al., 2014). Deletion of the Nef gene from simian immunodeficiency virus (SIV) and infection of rhesus monkeys with that virus showed lower viral load than the control group (Kestier et al., 1991). Similar experiments with mice that were transplanted with human tissues, exhibited slower progress in disease of mice infected with Nef-mutant HIV (Jamieson et al., 1994). Since Nef exerts its function in a non-enzymatic way, it is capable of modulating cellular signaling by binding to proteins of the host cell (Renkema and Saksela, 2000). Although Nef can interact with several molecules in the host cell, its activation of SFKs has been studied extensively and provides evidence that this interaction is in part responsible for the rapid progress in disease induced by Nef (Hanna et al., 2001; Trible et al., 2006; Narute and Smithgall, 2012; Alvarado et al., 2014). Also, Nef is able to downregulate major histocompatibility complex class I (MHC-I) on host cells (Collins et al., 1998). Notably, the binding of Nef to SFKs takes place between the SH3 domain of SFKs and a proline-rich region near the N-terminal of the Nef protein (PXXP) (Chi-Hon et al., 1996; Trible et al., 2006; Alvarado et al., 2014). Mutations in the PXXP motif resulted in the disappearance of Nef’s effects on the host cells (Hanna et al., 2001). In particular, Hck is the SFK that binds to Nef with the highest affinity and constitutes an important step in Nef signaling (Saksela et al., 1995; Briggs et al., 1997; Hanna et al., 2001). Moreover, the effect of Nef on the SFK Lck is being debated, because some study groups reported an activating effect, whereas an inhibitory effect was observed by other study groups (Greenway et al., 1996; Del Río-Iñiguez et al., 2018). Targeting SFKs, in particular Hck, might have potential for HIV/AIDS treatment (Amata et al., 2014). Indeed, CSK was able to inhibit or at least reduce tyrosine phosphorylation by SFKs in HIV-1 Nef infected yeast cells, except for Nef from the HIV-1 strain SF2 (Narute and Smithgall, 2012). However, Trible et al. reported that Nef-induced SFK activity is able to induce yeast cell growth even though CSK is expressed in these cells (Trible et al., 2006). Hence, myeloid cells potentially might miss sufficient CSK to counteract SFK activation through Nef (Briggs et al., 2001).

## Conclusion

The tyrosine protein kinase CSK is able to efficiently inhibit SFKs *in vitro* and *in vivo* (Hui and Vale, 2014). SFK regulation mainly occurs through phosphorylation of conserved tyrosine residues of the protein. Phosphorylation of the C-terminal regulatory tyrosine, as performed by CSK, inhibits SFK activity, whereas phosphorylation of tyrosine residues in the activation loop enhances SFK activity. Simultaneously, the dephosphorylation on these respective sites has an opposing effect on SFKs. Although CSK and SFKs resemble in protein structure regarding their domains, the lack of a C-terminal regulatory tyrosine of CSK should be emphasized on the one hand, and of the N-terminal acylation on the other hand, making CSK a cytoplasmic protein, unlike SFKs that are membrane-bound. This difference in cellular location especially presents an obstacle for CSK activity, as other recruiter proteins such as PAG/Cbp, LIME, paxillin and Dok-1 and Dok-2 are needed to enable spatial proximity of CSK with SFKs. SFKs regulate a great variety of cellular functions, by multiple signaling cascades, that include MAPK/ERK, STAT3, PI3K/AKT, VEGF, IL-8 pathways. While active, SFKs promote cell survival, proliferation, angiogenesis and cytoskeletal reorganization amongst others. SFK suppression by CSK in different settings most likely induces apoptosis and inhibits uncontrolled cell growth and migration. Thus, CSK has a pro-apoptotic effect by inhibiting activation of SFKs, which, in an active state, would lead to the downstream activation of MAPK/ERK, STAT3 and PI3K/AKT signaling pathways. By inhibiting the latter, AKT-mediated inhibition of FOXO can cease, and FOXO is hence potentially able to exert its pro-apoptotic effect by regulating the transcription of its target genes in the nucleus. As a result, apoptotic pathways can get activated leading to the activation of effector caspases that induce the molecular and structural changes for cell death.

At the same time, short SFK activation followed by CSK-mediated SFK inhibition controls actin reorganization required for apoptosis as it ensures the morphologic cellular changes during apoptosis, resulting in the fragmentation of the cell into apoptotic bodies.

The clinical importance of SFKs and CSK is underlined by the various diseases they have been associated with. These include cancer, autoinflammatory, autoimmune and neurologic diseases, hypertension and HIV/AIDS.

Indeed, inhibitors of SFKs such as dasatinib, PP2, saracatinib and SI221 have already been approved for treating different types of hematologic cancers, such as chronic myelogenous leukemia (U.S. Food and Drug Administration, 2017) and acute lymphoblastic leukemia (Drugs.com, 2019) in both adult and pediatric patients. Their uses have also been discussed in treating solid tumors, with positive effects obtained by Redin et al. by administering dasatinib alongside immune checkpoint inhibitors in NSCLC which accelerated tumor regression, while clinical studies are also in progress regarding the use of SFK inhibitors, such as SI221, for treating rhabdomyosarcoma (Redin et al., 2021). SI221 is a pyrazolo-pyrimidine derivative which exerts inhibitory effects on Yes (a member of SFKs) which is overexpressed in rhabdomyosarcoma, thus reducing proliferation of cancer cells (Bagella and Marchesi, 2016). Other such molecules have been studied for treating different types of solid tumors, namely SAB298, which has shown promising effects of tumor growth inhibition *in vivo* for patients with melanoma. However, clinical trials need to be performed in order to certify its utility (Halaban et al., 2019).

Furthermore, the linkage of CSK to a wide variety of diseases might have potential to be used in the clinical field, as the search for potent CSK inhibitors/activators is ongoing (O’Malley, 2020). Until now, pyridazinone and pyrazolopyridine have proved efficient in significantly reducing SFK phosphorylation by CSK *in vivo* (O’Malley et al., 2019). In this study, the substances were then tested in T-cells and produced greater T-cell activation in response to a stimulating antigen. Thus, the optimization and application of pyridazinone and pyrazolopyridine could be promising for cancer immunotherapy. The challenge in the development of such a CSK inhibitor relies in obtaining substrate specificity of the agent to CSK, an objective that is made difficult by the structural resemblance of CSK and SFKs (O’Malley, 2020). Hence, more research is needed to further investigate the implication of CSK in these various diseases, its potential as a target for therapy and the influence this might have on cell survival and apoptosis.

## References

[B1] Ahmed S. N. Jhaj R. Sadasivam B. Joshi R. (2020). Regression of the left ventricular hypertrophy in patients with essential hypertension on standard Drug therapy. Discoveries 8 (3), e138. 10.15190/D.2021.17 PMC7575414 33102689

[B2] Ahmed S. N. Jhaj R. Sadasivam B. Joshi R. (2021). Reversal of hypertensive heart disease: A multiple linear regression model. Discoveries 9 (4), e115. 10.15190/d.2020.12 PMC8898147 35261921

[B3] Aitken R. J. Baker M. A. (2013). Causes and consequences of apoptosis in spermatozoa; contributions to infertility and impacts on development. Int. J. Dev. Biol. 57, 265–272. 10.1387/IJDB.130146JA 23784837

[B4] Alvarado J. J. Tarafdar S. Yeh J. I. Smithgall T. E. (2014). Interaction with the Src homology (SH3-SH2) region of the Src-family kinase Hck structures the HIV-1 Nef dimer for kinase activation and effector recruitment. J. Biol. Chem. 289, 28539–28553. 10.1074/JBC.M114.600031 25122770 PMC4192505

[B5] Amata I. Maffei M. Pons M. (2014). Phosphorylation of unique domains of Src family kinases. Front. Genet. 5, 181. 10.3389/fgene.2014.00181 25071818 PMC4075076

[B6] Anwar H. Khan Q. U. Nadeem N. Pervaiz I. Ali M. Cheema F. F. (2020). Epileptic seizures. Discoveries 8 (2), e110. 10.15190/D.2020.7 32577498 PMC7305811

[B134] Bagella L. Marchesi I. (2016). SFK inhibitors as new strategy for RMS treatment. Chemotherapy 5 (2), 1–2. 10.4172/2167-7700.1000189

[B7] Baumeister U. Funke R. Ebnet K. Vorschmitt H. Koch S. Vestweber D. (2005). Association of Csk to VE-cadherin and inhibition of cell proliferation. EMBO J. 24, 1686–1695. 10.1038/SJ.EMBOJ.7600647 15861137 PMC1142580

[B8] Bharti S. Inoue H. Bharti K. Hirsch D. S. Nie Z. Yoon H.-Y. (2007). Src-dependent phosphorylation of ASAP1 regulates podosomes. Mol. Cell. Biol. 27, 8271–8283. 10.1128/MCB.01781-06 17893324 PMC2169185

[B9] Brdičková N. Brdička T. Angelisová P. Horváth O. Špička J. Hilgert I. (2003). Lime: A new membrane raft-associated adaptor protein involved in CD4 and CD8 coreceptor signaling. J. Exp. Med. 198, 1453–1462. 10.1084/JEM.20031484 14610046 PMC2194115

[B10] Brian B. F. Freedman T. S. (2021). The src-family kinase Lyn in immunoreceptor signaling. Endocrinology 162 (10), bqab152. 10.1210/ENDOCR/BQAB152 34320188 PMC8389176

[B11] Briggs S. D. Scholtz B. Jacque J. M. Swingler S. Stevenson M. Smithgall T. E. (2001). HIV-1 nef promotes survival of myeloid cells by a stat3-dependent pathway. J. Biol. Chem. 276, 25605–25611. 10.1074/jbc.M103244200 11328823 PMC9486509

[B12] Briggs S. D. Sharkey M. Stevenson M. Smithgall T. E. (1997). SH3-mediated Hck tyrosine kinase activation and fibroblast transformation by the Nef protein of HIV-1. J. Biol. Chem. 272, 17899–17902. 10.1074/jbc.272.29.17899 9218412

[B13] Briner A. Götz J. Polanco J. C. (2020). Fyn kinase controls tau aggregation *in vivo* . Cell Rep. 32, 108045. 10.1016/J.CELREP.2020.108045 32814048

[B14] Bucur O. Gaidos G. Yatawara A. Pennarun B. Rupasinghe C. Roux J. (2015). A novel caspase 8 selective small molecule potentiates TRAIL-induced cell death. Sci. Rep. 5, 9893. 10.1038/SREP09893 25962125 PMC4426715

[B15] Bucur O. (2016). microRNA regulators of apoptosis in cancer. Discoveries 4 (1), e57. 10.15190/d.2016.4 32309578 PMC7159826

[B16] Bucur O. Nat R. Cretoiu D. Popescu L. M. (2001). Phagocytosis of apoptotic cells by microglia *in vitro* . J. Cell. Mol. Med. 5, 438–441. 10.1111/J.1582-4934.2001.TB00181.X 12067480 PMC6740213

[B17] Bucur O. Ray S. Bucur M. C. Almasan A. (2006). APO2 ligand/tumor necrosis factor-related apoptosis-inducing ligand in prostate cancer therapy. Front. Biosci. 11, 1549–1568. 10.2741/1903 16368536

[B18] Bucur O. Stancu A. L. Muraru M. S. Melet A. Petrescu S. M. Khosravi-Far R. (2014). PLK1 is a binding partner and a negative regulator of FOXO3 tumor suppressor. Discoveries 2, e16. 10.15190/D.2014.8 26280018 PMC4535815

[B19] Cao M. Peng B. Chen H. Yang M. Chen P. Ye L. (2022). miR-34a induces neutrophil apoptosis by regulating Cdc42-WASP-Arp2/3 pathway-mediated F-actin remodeling and ROS production. Redox Rep. 27, 167–175. 10.1080/13510002.2022.2102843 35938579 PMC9364709

[B135] Chang F. Lemmon C. Lietha D. Eck M. Romer L. (2011). Tyrosine phosphorylation of Rac1: A role in regulation of cell spreading. PLoS One 6, e28587. 10.1371/journal.pone.0028587 22163037 PMC3232246

[B20] Chen C. Y. Fang H. Y. Chiou S. H. Yi S. E. Huang C. Y. Chiang S. F. (2011). Sumoylation of eukaryotic elongation factor 2 is vital for protein stability and anti-apoptotic activity in lung adenocarcinoma cells. Cancer Sci. 102, 1582–1589. 10.1111/J.1349-7006.2011.01975.X 21554491 PMC11159786

[B21] Chen J. Elfiky A. Han M. Chen C. Saif M. W. (2014). The role of src in colon cancer and its therapeutic implications. Clin. Colorectal Cancer 13, 5–13. 10.1016/J.CLCC.2013.10.003 24361441

[B22] Chi-Hon L. Saksela K. Mirza U. A. Chait B. T. Kuriyan J. (1996). Crystal structure of the conserved core of HIV-1 Nef complexed with a Src family SH3 domain. Cell 85, 931–942. 10.1016/S0092-8674(00)81276-3 8681387

[B23] Chin J. Palop J. J. Puoliväli J. Massaro C. Bien-Ly N. Gerstein H. (2005). Fyn kinase induces synaptic and cognitive impairments in a transgenic mouse model of Alzheimer’s disease. J. Neurosci. 25, 9694–9703. 10.1523/JNEUROSCI.2980-05.2005 16237174 PMC6725734

[B24] Chong Y. P. Ia K. K. Mulhern T. D. Cheng H. C. (2005). Endogenous and synthetic inhibitors of the Src-family protein tyrosine kinases. Biochim. Biophys. Acta 1754, 210–220. 10.1016/J.BBAPAP.2005.07.027 16198159

[B25] Chong Y. P. Mulhern T. D. Cheng H. C. (2009). C-Terminal src kinase (CSK) and CSK-homologous kinase (CHK)—Endogenous negative regulators of src-family protein kinases. Growth Factors. 23. 233-44. 10.1080/08977190500178877 16243715

[B26] Cloutier J. F. Veillette A. (1996). Association of inhibitory tyrosine protein kinase p50csk with protein tyrosine phosphatase PEP in T cells and other hemopoietic cells. EMBO J. 15, 4909–4918. 10.1002/j.1460-2075.1996.tb00871.x 8890164 PMC452228

[B27] Cloutier J. F. Veillette A. (1999). Cooperative inhibition of T-cell antigen receptor signaling by a complex between a kinase and a phosphatase. J. Exp. Med. 189, 111–121. 10.1084/JEM.189.1.111 9874568 PMC1887684

[B28] Coleman M. L. Sahai E. A. Yeo M. Bosch M. Dewar A. Olson M. F. (2001). Membrane blebbing during apoptosis results from caspase-mediated activation of ROCK I. Nat. Cell Biol. 3 (4 3), 339–345. 10.1038/35070009 11283606

[B29] Collins K. L. Chen B. K. Kalams S. A. Walker B. D. Baltimore D. (1998). HIV-1 Nef protein protects infected primary cells against killing by cytotoxic T lymphocytes. Nature 391, 397–401. 10.1038/34929 9450757

[B136] Cooke M. Baker M. J. Kazanietz M. G. (2020). Rac-GEF/Rac signaling and metastatic dissemination in lung cancer. Front. Cell Dev. Biol. 8, 18. 10.3389/fcell.2020.00118 32158759 PMC7051914

[B30] Cui N. Liu T. Guo Y. Dou J. Yang Q. Zhang H. (2019). SUMOylation of csk negatively modulates its tumor suppressor function. Neoplasia 21, 676–688. 10.1016/J.NEO.2019.04.010 31125786 PMC6531875

[B137] Dai H. Lv Y. F. Yan G. N. Meng G. Zhang X. Guo Q. N. (2016). RanBP9/TSSC3 complex cooperates to suppress anoikis resistance and metastasis via inhibiting Src-mediated Akt signaling in osteosarcoma. Cell Death Dis. 7, e2572. 10.1038/cddis.2016.436 28032865 PMC5261021

[B31] Davidson D. Bakinowski M. Thomas M. L. Horejsi V. Veillette A. (2003). Phosphorylation-dependent regulation of T-cell activation by PAG/Cbp, a lipid raft-associated transmembrane adaptor. Mol. Cell. Biol. 23, 2017–2028. 10.1128/MCB.23.6.2017-2028.2003 12612075 PMC149484

[B32] Davidson D. Cloutier J. F. Gregorieff A. Veillette A. (1997). Inhibitory tyrosine protein kinase p50csk is associated with protein-tyrosine phosphatase PTP-PEST in hemopoietic and non-hemopoietic cells. J. Biol. Chem. 272, 23455–23462. 10.1074/JBC.272.37.23455 9287362

[B33] De Bhailis Á. M. Kalra P. A. (2022). Hypertension and the kidneys. Br. J. Hosp. Med. 83, 1–11. 10.12968/HMED.2021.0440 35653320

[B34] Del Río-Iñiguez I. Vázquez-Chávez E. Cuche C. di Bartolo V. Bouchet J. Alcover A. (2018). HIV-1 nef hijacks Lck and Rac1 endosomal traffic to dually modulate signaling-mediated and actin cytoskeleton–mediated T cell functions. J. Immunol. 201, 2624–2640. 10.4049/jimmunol.1800372 30282749

[B35] Dey N. De P. K. Wang M. Zhang H. Dobrota E. A. Robertson K. A. (2007). CSK controls retinoic acid receptor (RAR) signaling: A RAR-c-SRC signaling axis is required for neuritogenic differentiation. Mol. Cell. Biol. 27, 4179–4197. 10.1128/MCB.01352-06 17325034 PMC1900023

[B36] Dey N. Howell B. W. De P. K. Durden D. L. (2005). CSK negatively regulates nerve growth factor induced neural differentiation and augments AKT kinase activity. Exp. Cell Res. 307, 1–14. 10.1016/J.YEXCR.2005.02.029 15890337

[B37] Dodd D. A. Worth R. G. Rosen M. K. Grinstein S. van Oers N. S. C. Hansen E. J. (2014). The Haemophilus ducreyi LspA1 protein inhibits phagocytosis by using a new mechanism involving activation of C-terminal Src kinase. mBio 5 (3), e01178-14. 10.1128/MBIO.01178-14 24902122 PMC4030455

[B38] Drobek A. Kralova J. Skopcova T. Kucova M. Novák P. Angelisová P. (2015). PSTPIP2, a protein associated with autoinflammatory disease, interacts with inhibitory enzymes SHIP1 and csk. J. Immunol. 195, 3416–3426. 10.4049/jimmunol.1401494 26304991

[B39] Drugs.com (2019). Sprycel (dasatinib) tablets now approved in combination with chemotherapy in certain pediatric patients with philadelphia chromosome-positive acute lymphoblastic leukemia. (Accessed November 20, 2022).

[B40] Dumitrascu G. R. Bucur O. (2013). Critical physiological and pathological functions of Forkhead Box O tumor suppressors. Discoveries 1, e5. 10.15190/D.2013.5 32309538 PMC6941590

[B41] Elmore S. (2007). Apoptosis: A review of programmed cell death. Toxicol. Pathol. 35, 495–516. 10.1080/01926230701320337 17562483 PMC2117903

[B42] Engen J. R. Wales T. E. Hochrein J. M. Meyn M. A. Banu Ozkan S. Bahar I. (2008). Structure and dynamic regulation of src-family kinases. Cell. Mol. Life Sci. 65, 3058–3073. 10.1007/S00018-008-8122-2 18563293 PMC9357288

[B43] Fang X. Zhou X. Wang X. (2013). Clinical development of phosphatidylinositol 3-kinase inhibitors for non-Hodgkin lymphoma. Biomark. Res. 1, 30. 10.1186/2050-7771-1-30 24252186 PMC4177547

[B138] Feig C. Peter M. E. (2007). How apoptosis got the immune system in shape. Eur. J. Immunol. 37 (Suppl 1). 10.1002/eji.200737462 17972347

[B44] Fortner A. Bucur O. (2022). mRNA-based vaccine technology for HIV. Discoveries 10 (2), e150. 10.15190/d.2022.9 36438441 PMC9683993

[B45] Franklin-Tong V. E. Gourlay C. W. (2008). A role for actin in regulating apoptosis/programmed cell death: Evidence spanning yeast, plants and animals. Biochem. J. 413, 389–404. 10.1042/BJ20080320 18613816

[B46] Gasteier J. E. Madrid R. Krautkrämer E. Schröder S. Muranyi W. Benichou S. (2003). Activation of the Rac-binding partner FHOD1 induces actin stress fibers via a ROCK-dependent mechanism. J. Biol. Chem. 278, 38902–38912. 10.1074/jbc.M306229200 12857739

[B47] Gasteier J. E. Schroeder S. Muranyi W. Madrid R. Benichou S. Fackler O. T. (2005). FHOD1 coordinates actin filament and microtubule alignment to mediate cell elongation. Exp. Cell Res. 306 (1), 192–202. 10.1016/j.yexcr.2005.02.006 15878344

[B139] Gerbec Z. J. Thakar M. S. Malarkannan S. (2015). The Fyn-ADAP axis: Cytotoxicity versus cytokine production in killer cells. Front. Immunol. 6, 472. 10.3389/fimmu.2015.00472 26441977 PMC4584950

[B48] Gomez-Sanchez C. E. Sapiro D. R. May K. v. Rainey W. E. Nishimoto K. Gomez-Sanchez E. P. (2022). Origin of circulating 18-oxocortisol in the normal human adrenal. Mol. Cell. Endocrinol. 555, 111720. 10.1016/J.MCE.2022.111720 35870737 PMC10911085

[B49] Grant S. G. N. O’Dell T. J. Karl K. A. Stein P. L. Soriano P. Kandel E. R. (1992). Impaired long-term potentiation, spatial learning, and hippocampal development in fyn mutant mice. Science 258, 1903–1910. 10.1126/SCIENCE.1361685 1361685

[B50] Greenway A. Azad A. Mills J. McPhee D. (1996). Human immunodeficiency virus type 1 Nef binds directly to Lck and mitogen-activated protein kinase, inhibiting kinase activity. J. Virol. 70, 6701–6708. 10.1128/JVI.70.10.6701-6708.1996 8794306 PMC190712

[B51] Guarino M. (2010). Src signaling in cancer invasion. J. Cell. Physiol. 223, 14–26. 10.1002/JCP.22011 20049846

[B52] Guo Y.-J. Pan W.-W. Liu S.-B. Shen Z.-F. Xu Y. Hu L.-L. (2020). ERK/MAPK signalling pathway and tumorigenesis. Exp. Ther. Med. 19, 1997–2007. 10.3892/ETM.2020.8454 32104259 PMC7027163

[B53] Hanna Z. Weng X. Kay D. G. Poudrier J. Lowell C. Jolicoeur P. (2001). The pathogenicity of human immunodeficiency virus (HIV) type 1 nef in CD4C/HIV transgenic mice is abolished by mutation of its SH3-binding domain, and disease development is delayed in the absence of Hck. J. Virol. 75, 9378–9392. 10.1128/JVI.75.19.9378-9392.2001 11533201 PMC114506

[B54] Hannemann S. Madrid R. Stastna J. Kitzing T. Gasteier J. Schönichen A. (2008). The diaphanous-related formin FHOD1 associates with ROCK1 and promotes src-dependent plasma membrane blebbing. J. Biol. Chem. 283, 27891–27903. 10.1074/JBC.M801800200 18694941

[B55] Harris M. (1999). HIV: A new role for nef in the spread of HIV. Curr. Biol. 9, R459–R461. 10.1016/S0960-9822(99)80282-6 10375524

[B56] Hartmann S. Ridley A. J. Lutz S. (2015). The function of rho-associated kinases ROCK1 and ROCK2 in the pathogenesis of cardiovascular disease. Front. Pharmacol. 6, 276. 10.3389/fphar.2015.00276 26635606 PMC4653301

[B57] Ho H. Y. Rohatgi R. Lebensohn A. M. Ma L. Li J. Gygi S. P. (2004). Toca-1 mediates Cdc42-dependent actin nucleation by activating the N-WASP-WIP complex. Cell 118, 203–216. 10.1016/j.cell.2004.06.027 15260990

[B58] Hui E. Vale R. D. (2014). *In vitro* membrane reconstitution of the T-cell receptor proximal signaling network. Nat. Struct. Mol. Biol. 21, 133–142. 10.1038/NSMB.2762 24463463 PMC4062301

[B140] Ia K. K. Mills R. D. Hossain M. I. Chan K. C. Jarasrassamee B. Jorissen R. N. (2010). Structural elements and allosteric mechanisms governing regulation and catalysis of CSK-family kinases and their inhibition of Src-family kinases. Growth Factors 28, 329–350. 10.3109/08977194.2010.484424 20476842

[B59] Imamoto A. Soriano P. (1993). Disruption of the csk gene, encoding a negative regulator of Src family tyrosine kinases, leads to neural tube defects and embryonic lethality in mice. Cell 73, 1117–1124. 10.1016/0092-8674(93)90641-3 7685657

[B60] Ishizawar R. Parsons S. J. (2004). C-Src and cooperating partners in human cancer. Cancer Cell 6, 209–214. 10.1016/j.ccr.2004.09.001 15380511

[B61] Jamieson B. D. Aldrovandi G. M. Planelles V. Jowett J. B. Gao L. Bloch L. M. (1994). Requirement of human immunodeficiency virus type 1 nef for *in vivo* replication and pathogenicity. J. Virol. 68, 3478–3485. 10.1128/JVI.68.6.3478-3485.1994 8189487 PMC236850

[B62] Jiang L. Q. Feng X. Zhou W. Knyazev P. G. Ullrich A. Chen Z. (2006). Csk-binding protein (Cbp) negatively regulates epidermal growth factor-induced cell transformation by controlling Src activation. Oncogene 25, 5495–5506. 10.1038/sj.onc.1209554 16636672

[B141] Kazanietz M. G. Cooke M. Garcia-Mata R. (2022). Nonredundant Rac-GEF control of actin cytoskeleton reorganization. Trends Cell Biol. 32, 815–818. 10.1016/j.tcb.2022.06.003 35753960 PMC9930409

[B63] Kestier H. W. Ringler D. J. Mori K. Panicali D. L. Sehgal P. K. Daniel M. D. (1991). Importance of the nef gene for maintenance of high virus loads and for development of AIDS. Cell 65, 651–662. 10.1016/0092-8674(91)90097-I 2032289

[B64] Kim L. C. Song L. Haura E. B. (2009). Src kinases as therapeutic targets for cancer. Nat. Rev. Clin. Oncol. 6 (10 6), 587–595. 10.1038/nrclinonc.2009.129 19787002

[B83] Kim S.M. Kang J.-O. Lim J. E. Hwang S. Y. Oh B. (2017). Csk regulates blood pressure by controlling the synthetic pathways of aldosterone. Circ. J. 82 (1), 168–175. 10.1253/circj.CJ-17-0080 28724838

[B65] Knox R. Jiang X. (2015). Fyn in neurodevelopment and ischemic brain injury. Dev. Neurosci. 37, 311–320. 10.1159/000369995 25720756 PMC4713834

[B66] Kojima N. Ishibashi H. Obata K. Kandel E. R. (1998). Higher seizure susceptibility and enhanced tyrosine phosphorylation of N-methyl-D-aspartate receptor subunit 2B in fyn transgenic mice. Learn. Mem. 5 (6), 429–444. 10489260 PMC311255

[B67] Kumar R. Herbert P. E. Warrens A. N. (2005). An introduction to death receptors in apoptosis. Int. J. Surg. 3, 268–277. 10.1016/J.IJSU.2005.05.002 17462297

[B68] Lavoie J. N. Landry M. C. Faure R. L. Champagne C. (2010). Src-family kinase signaling, actin-mediated membrane trafficking and organellar dynamics in the control of cell fate: Lessons to be learned from the adenovirus E4orf4 death factor. Cell. Signal. 22, 1604–1614. 10.1016/J.CELLSIG.2010.04.007 20417707

[B69] Lee H. J. Kang J. O. Kim S. M. Ji S. M. Park S. Y. Kim M. E. (2016). Gene silencing and haploinsufficiency of csk increase blood pressure. PLoS One 11, e0146841. 10.1371/JOURNAL.PONE.0146841 26751575 PMC4713444

[B70] Lee H. Volonte D. Galbiati F. Iyengar P. Lublin D. M. Bregman D. B. (2000). Constitutive and growth factor-regulated phosphorylation of caveolin-1 occurs at the same site (Tyr-14) *in vivo*: Identification of a c-src/cav-1/grb7 signaling cassette. Mol. Endocrinol. 14, 1750–1775. 10.1210/MEND.14.11.0553 11075810

[B71] Li P. Dong X. R. Zhang B. Zhang X. T. Liu J. Z. Ma D. S. (2021). Molecular mechanism and therapeutic targeting of necrosis, apoptosis, pyroptosis, and autophagy in cardiovascular disease. Chin. Med. J. 134, 2647–2655. 10.1097/CM9.0000000000001772 34608069 PMC8631411

[B72] Lin X. Lee S. Sun G. (2003). Functions of the activation loop in csk protein-tyrosine kinase. J. Biol. Chem. 278, 24072–24077. 10.1074/JBC.M210596200 12686554

[B73] Lindquist S. Karitkina D. Langnaese K. Posevitz-Fejfar A. Schraven B. Xavier R. (2011). Phosphoprotein associated with glycosphingolipid-enriched microdomains differentially modulates SRC kinase activity in brain maturation. PLoS One 6 (9), e23978. 10.1371/JOURNAL.PONE.0023978 21915273 PMC3167820

[B75] Liu S. Xiong X. Zhao X. Yang X. Wang H. (2015). F-BAR family proteins, emerging regulators for cell membrane dynamic changes - from structure to human diseases. J. Hematol. Oncol. 8, 47–14. 10.1186/s13045-015-0144-2 25956236 PMC4437251

[B142] Liu T. Han S. Dai Q. Zheng J. Liu X. Li S. (2019). IL-17A-mediated excessive autophagy aggravated neuronal ischemic injuries via Src-PP2B-mTOR pathway. Front. Immunol. 10, 2952. 10.3389/fimmu.2019.02952 31921197 PMC6933613

[B74] Liu G. Fiock K. L. Levites Y. Golde T. E. Hefti M. M. Lee G. (2020). Fyn depletion ameliorates tauP301L-induced neuropathology. Acta Neuropathol. Commun. 8, 108. 10.1186/S40478-020-00979-6 32665013 PMC7362472

[B76] Ma J. H. Qin L. Li X. (2020). Role of STAT3 signaling pathway in breast cancer. Cell Commun. Signal. 18, 33. 10.1186/S12964-020-0527-Z 32111215 PMC7048131

[B77] Manjarrez-Orduño N. Marasco E. Chung S. A. Katz M. S. Kiridly J. F. Simpfendorfer K. R. (2012). CSK regulatory polymorphism is associated with systemic lupus erythematosus and influences B cell signaling and activation. Nat. Genet. 44, 1227–1230. 10.1038/NG.2439 23042117 PMC3715052

[B78] Manz B. N. Tan Y. X. Courtney A. H. Rutaganira F. Palmer E. Shokat K. M. (2015). Small molecule inhibition of Csk alters affinity recognition by T cells. Elife 4, e08088. 10.7554/ELIFE.08088 26302204 PMC4568592

[B79] Masaki T. Okada M. Tokuda M. Shiratori Y. Hatase O. Shirai M. (1999). Reduced C-terminal Src kinase (Csk) activities in hepatocellular carcinoma. Hepatology 29, 379–384. 10.1002/HEP.510290239 9918913

[B80] Nada S. Yagi T. Takeda H. Tokunaga T. Nakagawa H. Ikawa Y. (1993). Constitutive activation of Src family kinases in mouse embryos that lack Csk. Cell 73, 1125–1135. 10.1016/0092-8674(93)90642-4 8513497

[B81] Narute P. S. Smithgall T. E. (2012). Nef alleles from all major HIV-1 clades activate src-family kinases and enhance HIV-1 replication in an inhibitor-sensitive manner. PLoS One 7. e32561, 10.1371/journal.pone.0032561 22393415 PMC3290594

[B82] Ogawa A. Takayama Y. Sakai H. Chong K. T. Takeuchi S. Nakagawa A. (2002). Structure of the carboxyl-terminal src kinase, csk. J. Biol. Chem. 277, 14351–14354. 10.1074/jbc.C200086200 11884384

[B84] Okada M. (2012). Regulation of the SRC family kinases by Csk. Int. J. Biol. Sci. 8, 1385–1397. 10.7150/IJBS.5141 23139636 PMC3492796

[B85] O’Malley D. P. Ahuja V. Fink B. Cao C. Wang C. Swanson J. (2019). Discovery of pyridazinone and pyrazolo[1, 5- a]pyridine inhibitors of C-terminal src kinase. ACS Med. Chem. Lett. 10, 1486–1491. 10.1021/acsmedchemlett.9b00354 31620238 PMC6792176

[B86] O’Malley D. P. (2020). Recent advances in inhibitors of C-terminal SRC kinase. Future Med. Chem. 12, 1447–1449. 10.4155/FMC-2020-0125 32638626

[B87] Oneyama C. Hikita T. Enya K. Dobenecker M. W. Saito K. Nada S. (2008). The lipid raft-anchored adaptor protein Cbp controls the oncogenic potential of c-src. Mol. Cell 30, 426–436. 10.1016/j.molcel.2008.03.026 18498747

[B88] Ortiz M. A. Mikhailova T. Li X. Porter B. A. Bah A. Kotula L. (2021). Src family kinases, adaptor proteins and the actin cytoskeleton in epithelial-to-mesenchymal transition. Cell Commun. Signal. 19 (1 19), 67–19. 10.1186/S12964-021-00750-X 34193161 PMC8247114

[B89] Park S. S. Eom Y. W. Kim E. H. Lee J. H. Min D. S. Kim S. (2004). Involvement of c-Src kinase in the regulation of TGF-beta1-induced apoptosis. Oncogene 23, 6272–6281. 10.1038/SJ.ONC.1207856 15208664

[B90] Pedraza L. G. Stewart R. A. Li D. M. Xu T. (2004). Drosophila Src-family kinases function with Csk to regulate cell proliferation and apoptosis. Oncogene 23, 4754–4762. 10.1038/SJ.ONC.1207635 15107833

[B91] Pennarun B. Gaidos G. Bucur O. Tinari A. Rupasinghe C. Jin T. (2013). killerFLIP: a novel lytic peptide specifically inducing cancer cell death. Cell Death Dis. 4, e894. 10.1038/CDDIS.2013.401 24176852 PMC3920952

[B92] Peterson J. R. Chernoff J. (2006). Src transforms in a Cool way. Nat. Cell Biol. 8, 905–907. 10.1038/ncb0906-905 16946737

[B93] Plati J. Bucur O. Khosravi-Far R. (2011). Apoptotic cell signaling in cancer progression and therapy. Integr. Biol. 3, 279–296. 10.1039/C0IB00144A PMC3130501 21340093

[B94] Plati J. Bucur O. Khosravi-Far R. (2008). Dysregulation of apoptotic signaling in cancer: Molecular mechanisms and therapeutic opportunities. J. Cell. Biochem. 104, 1124–1149. 10.1002/JCB.21707 18459149 PMC2941905

[B95] Potuckova L. Draberova L. Halova I. Paulenda T. Draber P. (2018). Positive and negative regulatory roles of C-terminal src kinase (CSK) in FcεRI-mediated mast cell activation, independent of the transmembrane adaptor PAG/CSK-binding protein. Front. Immunol. 9, 1771. 10.3389/fimmu.2018.01771 30116247 PMC6082945

[B96] Putra M. Puttachary S. Liu G. Lee G. Thippeswamy T. (2020). Fyn-tau ablation modifies PTZ-induced seizures and post-seizure hallmarks of early epileptogenesis. Front. Cell. Neurosci. 14, 428. 10.3389/fncel.2020.592374 PMC7752812 33363455

[B97] Rautureau Y. Paradis P. Schiffrin E. L. (2011). Cross-talk between aldosterone and angiotensin signaling in vascular smooth muscle cells. Steroids 76, 834–839. 10.1016/J.STEROIDS.2011.02.015 21371487

[B143] Redin E. Garmendia I. Lozano T. Serrano D. Senent Y. Redrado M. (2021). SRC family kinase (SFK) inhibitor dasatinib improves the antitumor activity of anti-PD-1 in NSCLC models by inhibiting Treg cell conversion and proliferation. J. Immunother. Cancer 9, e001496. 10.1136/jitc-2020-001496 33658304 PMC7931761

[B98] Reinehr R. Sommerfeld A. Häussinger D. (2013). The Src family kinases: Distinct functions of c-Src, Yes, and Fyn in the liver. Biomol. Concepts 4, 129–142. 10.1515/bmc-2012-0047 25436571

[B99] Renkema G. H. Saksela K. (2000). Interactions of HIV-1 NEF with cellular signal transducing proteins. Front. Biosci. 13, D268–D283. 10.2741/RENKEMA 10704155

[B100] Richardson E. T. Shukla S. Nagy N. Boom W. H. Beck R. C. Zhou L. (2015). ERK signaling is essential for macrophage development. PLoS One 10 (10), e0140064. 10.1371/journal.pone.0140064 26445168 PMC4596867

[B101] Robert A. Smadja-Lamère N. Landry M. C. Champagne C. Petrie R. Lamarche-Vane N. (2006). Adenovirus E4orf4 hijacks rho GTPase-dependent actin dynamics to kill cells: A role for endosome-associated actin assembly. Mol. Biol. Cell 17, 3329–3344. 10.1091/MBC.E05-12-1146 16687574 PMC1483059

[B102] Roskoski R. (2005). Src kinase regulation by phosphorylation and dephosphorylation. Biochem. Biophys. Res. Commun. 331, 1–14. 10.1016/J.BBRC.2005.03.012 15845350

[B103] Roskoski R. (2004). Src protein-tyrosine kinase structure and regulation. Biochem. Biophys. Res. Commun. 324, 1155–1164. 10.1016/J.BBRC.2004.09.171 15504335

[B104] Saito K. Enya K. Oneyama C. Hikita T. Okada M. (2008). Proteomic identification of ZO-1/2 as a novel scaffold for Src/Csk regulatory circuit. Biochem. Biophys. Res. Commun. 366, 969–975. 10.1016/J.BBRC.2007.12.055 18086565

[B105] Saksela K. Cheng G. Baltimore D. (1995). Proline-rich (PxxP) motifs in HIV-1 Nef bind to SH3 domains of a subset of Src kinases and are required for the enhanced growth of Nef+ viruses but not for down-regulation of CD4. EMBO J. 14, 484–491. 10.1002/J.1460-2075.1995.TB07024.X 7859737 PMC398106

[B106] Salter M. W. Kalia L. v. (2004). Src kinases: A hub for NMDA receptor regulation. Nat. Rev. Neurosci. 5 (4 5), 317–328. 10.1038/nrn1368 15034556

[B107] Samarasekera G. D. N. G. Auld V. J. (2018). C-terminal Src kinase (Csk) regulates the tricellular junction protein Gliotactin independent of Src. Mol. Biol. Cell 29, 123–136. 10.1091/MBC.E17-04-0251 29167383 PMC5909926

[B108] Satoh N. Nakamura M. Suzuki M. Suzuki A. Seki G. Horita S. (2015). Roles of akt and SGK1 in the regulation of renal tubular transport. Biomed. Res. Int. 2015, 971697. 10.1155/2015/971697 26491696 PMC4600925

[B109] Schaller M. D. Parsons J. T. (1995). pp125FAK-dependent tyrosine phosphorylation of paxillin creates a high-affinity binding site for Crk. Mol. Cell. Biol. 15, 2635–2645. 10.1128/MCB.15.5.2635 7537852 PMC230493

[B110] Singh A. Ye M. Bucur O. Zhu S. Santos M. T. Rabinovitz I. (2010). Protein phosphatase 2A reactivates FOXO3a through a dynamic interplay with 14-3-3 and AKT. Mol. Biol. Cell 21, 1140–1152. 10.1091/MBC.E09-09-0795 20110348 PMC2836964

[B111] Sirvent A. Bénistant C. Pannequin J. Veracini L. Simon V. Bourgaux J. F. (2009). Src family tyrosine kinases-driven colon cancer cell invasion is induced by Csk membrane delocalization. Oncogene 29 (9 29), 1303–1315. 10.1038/onc.2009.450 20010872

[B112] Sirvent A. Benistant C. Roche S. (2012). Oncogenic signaling by tyrosine kinases of the SRC family in advanced colorectal cancer. Am. J. Cancer Res. 2, 357–371. 22860228 PMC3410585

[B113] Sondhi D. Xu W. Songyang Z. Eck M. J. Cole P. A. (1998). Peptide and protein phosphorylation by protein tyrosine kinase csk: Insights into specificity and mechanism. Biochemistry 37, 165–172. 10.1021/BI9722960 9425036

[B114] Sun B. Zhong F. J. (2021). ELTD1 promotes gastric cancer cell proliferation, invasion and epithelial-mesenchymal transition through MAPK/ERK signaling by regulating CSK. Int. J. Gen. Med. 14, 4897–4911. 10.2147/IJGM.S325495 34475781 PMC8407680

[B115] Szilveszter K. P. Németh T. Mócsai A. (2019). Tyrosine kinases in autoimmune and inflammatory skin diseases. Front. Immunol. 10, 1862. 10.3389/fimmu.2019.01862 31447854 PMC6697022

[B116] Thomas S. M. Brugge J. S. (1997). Cellular functions regulated by Src family kinases. Annu. Rev. Cell Dev. Biol. 13, 513–609. 10.1146/ANNUREV.CELLBIO.13.1.513 9442882

[B117] Tizaoui K. Terrazzino S. Cargnin S. Lee K. H. Gauckler P. Li H. (2021). The role of PTPN22 in the pathogenesis of autoimmune diseases: A comprehensive review. Semin. Arthritis Rheum. 51, 513–522. 10.1016/J.SEMARTHRIT.2021.03.004 33866147

[B118] Tomaszewski M. Morris A. P. Howson J. M. M. Franceschini N. Eales J. M. Xu X. (2022). Kidney omics in hypertension: From statistical associations to biological mechanisms and clinical applications. Kidney Int. 102, 492–505. 10.1016/J.KINT.2022.04.045 35690124 PMC9886011

[B119] Torgersen K. M. Vang T. Abrahamsen H. Yaqub S. Hořejší V. Schraven B. (2001). Release from tonic inhibition of T cell activation through transient displacement of C-terminal Src kinase (Csk) from lipid rafts. J. Biol. Chem. 276, 29313–29318. 10.1074/JBC.C100014200 11390365

[B120] Touyz R. M. Wu X. H. He G. Salomon S. Schiffrin E. L. (2002). Increased angiotensin II-mediated Src signaling via epidermal growth factor receptor transactivation is associated with decreased C-terminal Src kinase activity in vascular smooth muscle cells from spontaneously hypertensive rats. Hypertension 39, 479–485. 10.1161/HY02T2.102909 11882594

[B121] Trepanier C. H. Jackson M. F. MacDonald J. F. (2012). Regulation of NMDA receptors by the tyrosine kinase Fyn. FEBS J. 279, 12–19. 10.1111/J.1742-4658.2011.08391.X 21985328

[B122] Trible R. P. Emert-Sedlak L. Smithgall T. E. (2006). HIV-1 Nef selectively activates Src family kinases Hck, Lyn, and c-Src through direct SH3 domain interaction. J. Biol. Chem. 281, 27029–27038. 10.1074/jbc.M601128200 16849330 PMC2892265

[B123] U.S. Food and Drug Administration (2017). FDA approves dasatinib for pediatric patients with CML. (Accessed November 20, 2022).

[B124] Valinsky W. C. Touyz R. M. Shrier A. (2019). Aldosterone and ion channels. Vitam. Horm. 109, 105–131. 10.1016/BS.VH.2018.10.004 30678852

[B125] Vidal S. Bouzaher Y. H. el Motiam A. Seoane R. Rivas C. (2021). Overview of the regulation of the class IA PI3K/AKT pathway by SUMO. Semin. Cell Dev. Biol. 132, 51–61. 10.1016/J.SEMCDB.2021.10.012 34753687

[B126] Wang B. Lemay S. Tsai S. Veillette A. (2001). SH2 domain-mediated interaction of inhibitory protein tyrosine kinase csk with protein tyrosine phosphatase-HSCF. Mol. Cell. Biol. 21, 1077–1088. 10.1128/MCB.21.4.1077-1088.2001 11158295 PMC99562

[B127] Watanabe N. Matsuda S. Kuramochi S. Tsuzuku J. Yamamoto T. Endo K. (1995). Expression of C-terminal src kinase in human colorectal cancer cell lines. Jpn. J. Clin. Oncol. 25 (1), 5–9. PMID: 7533218. 7533218

[B128] Watson J. R. Owen D. Mott H. R. (2017). Cdc42 in actin dynamics: An ordered pathway governed by complex equilibria and directional effector handover. Small GTPases 8, 237–244. 10.1080/21541248.2016.1215657 27715449 PMC5680673

[B129] Williamson R. Scales T. Clark B. R. Gibb G. Hugh Reynolds C. Kellie S. (2002). Rapid tyrosine phosphorylation of neuronal proteins including tau and focal adhesion kinase in response to amyloid-β peptide exposure: Involvement of src family protein kinases. J. Neurosci. 22, 10–20. 10.1523/JNEUROSCI.22-01-00010.2002 11756483 PMC6757621

[B130] Xu J. J. Li H. Du X. S. Li J. J. Meng X. M. Huang C. (2021). Role of the F-bar family member PSTPIP2 in autoinflammatory diseases. Front. Immunol. 12, 585412. 10.3389/fimmu.2021.585412 34262554 PMC8273435

[B131] Yagi R. Waguri S. Sumikawa Y. Nada S. Oneyama C. Itami S. (2007). C-terminal Src kinase controls development and maintenance of mouse squamous epithelia. EMBO J. 26, 1234–1244. 10.1038/SJ.EMBOJ.7601595 17304209 PMC1817640

[B132] Yao Q. Liu B. Q. Li H. McGarrigle D. Xing B. W. Zhou M. T. (2014). C-terminal src kinase (CSK)-mediated phosphorylation of eukaryotic elongation factor 2 (EEF2) promotes proteolytic cleavage and nuclear translocation of EEF2. J. Biol. Chem. 289, 12666–12678. 10.1074/jbc.M113.546481 24648518 PMC4007456

[B133] Yasuda T. Bundo K. Hino A. Honda K. Inoue A. Shirakata M. (2007). Dok-1 and Dok-2 are negative regulators of T cell receptor signaling. Int. Immunol. 19, 487–495. 10.1093/INTIMM/DXM015 17329234

[B144] Zhao G. S. Gao Z. R. Zhang Q. Tang X. F. Lv Y. F. Zhang Z. S. (2018). TSSC3 promotes autophagy via inactivating the Src-mediated PI3K/Akt/mTOR pathway to suppress tumorigenesis and metastasis in osteosarcoma, and predicts a favorable prognosis. J. Exp. Clin. Cancer Res. 37, 188. 10.1186/S13046-018-0856-6 30092789 PMC6085607

